# MYB80 and TEK: Dynamic duo regulating callose wall degradation and pollen exine development

**DOI:** 10.1093/plphys/kiaf147

**Published:** 2025-04-10

**Authors:** Nilesh D Gawande

**Affiliations:** Assistant Features Editor, Plant Physiology, American Society of Plant Biologists; Department of Biological Sciences and Engineering, Indian Institute of Technology Gandhinagar, Palaj 382355, Gujarat, India

Plant reproduction is a complex process that leads to the formation of male and female gametes in pollen and ovules, respectively. Pollen development starts with meiosis in the pollen mother cells, producing haploid pollen cell tetrads. Each tetrad is surrounded by an inner protective barrier, called the callose wall, and an outer layer made of cellulose and pectin. The callose wall, made of ß-1,3 glucan, is important for forming an outer pollen layer known as exine (Radja et al. 2019), which has two parts: the sexine (outer region) and the nexine (beneath it). The callose wall will break down later, releasing the microspores from the tetrads, which then mature further in locules. Thus, the formation and breakdown of the tetrad callose wall are critical for pollen development.

Callose synthases are crucial for callose formation during the development of the callose wall. In contrast, callase secreted by the tapetum (the innermost layer of the anther) are enzymes consisting of ß-1,3-glucanase, which help break down the callose wall and facilitate the release of microspores. Changes in the expression of β-1,3-glucanase affect the degradation of the callose wall, leading to issues in pollen development. Early expression of β-1,3-glucanase can cause premature breakdown of callose, resulting in pollen collapse in tobacco (Worrall et al. 1992). Thus, the breakdown of the tetrad wall by β-1,3-glucanase is also a critical step in developing the pollen wall.

**Figure 1.**
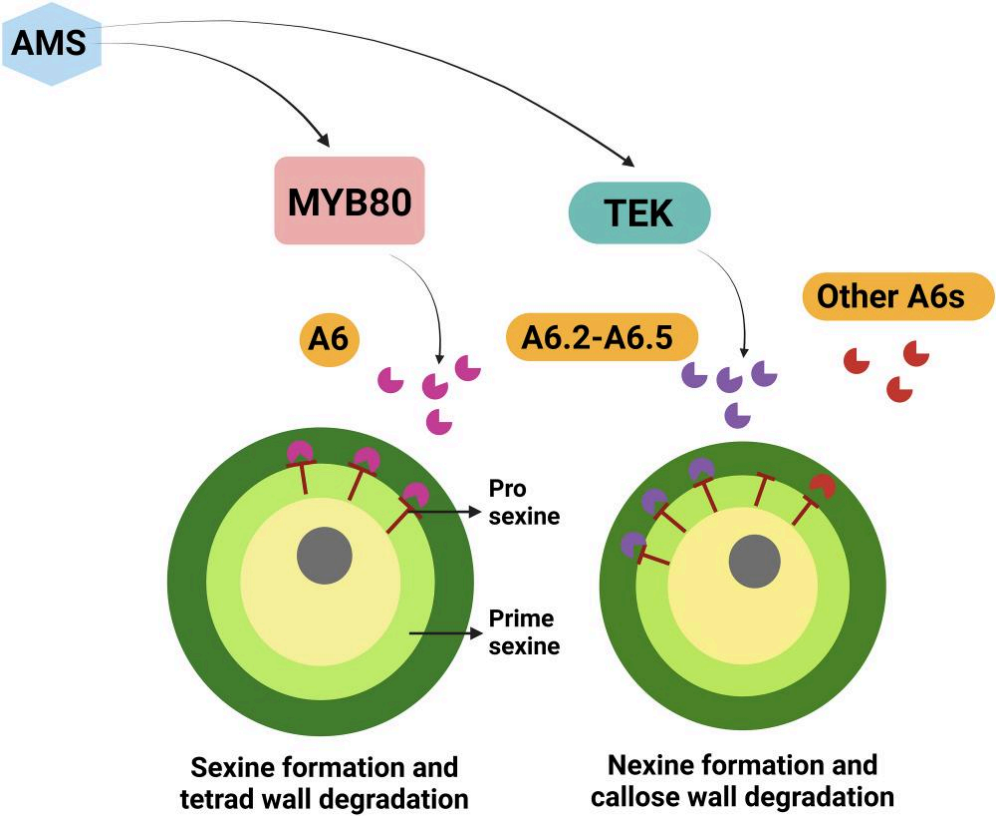
Callose wall degradation and pollen development through MYB80 and TEK in *Arabidopsis*. AMS regulates MYB80 for the formation of sexine, while TEK facilitates the development of nexine. MYB80 forms the T-shaped prosexine within primesexine and regulates the expression of A6 in the tapetum, which is subsequently secreted into the anther locule to promote callose degradation. Both MYB80 and TEK directly influence the expression of *A6*s (*A6*, *A6.2*-*A6.*5) and drive rapid callose degradation in the tapetum. Additionally, other A6s in the tapetum serve as a safeguard for the degradation of the callose wall. The figure is modified from Xu et al. (2025) and was created with BioRender.com.

*MYB80* encodes an R2R3 MYB transcription factor essential for the formation of sexine (Zhang et al. 2007), while *TEK* encodes another transcription factor involved in nexine formation (Lou et al. 2014). ABORTED MICROSPORE (AMS) is a basic helix-loop-helix factor that regulates exine formation by affecting the expression of *MYB80* and *TEK* in the tapetum (Lou et al. 2014) (Fig. 1). However, the specific roles of MYB80 and TEK in callose breakdown are still unclear. In both *Arabidopsis* and *Brassica napus*, the *A6* gene, which encodes a specific β-1,3-glucanase, is known to be involved in callose degradation (Fig. 1). Therefore, investigating the roles of MYB, TEK, and A6 in callose degradation and pollen development is an important area for research.

In this issue of *Plant Physiology*, Xu et al. (2025) investigated the role of MYB80 and TEK in degrading the callose tetrad wall and in regulating five *A6* genes. Microspores in the double mutants *myb80 tek* displayed a complete absence of both sexine and nexine. Staining assays for callose wall degradation indicated the presence of visible callose and a delayed release of microspores from tetrads in the *myb80 tek* anther compared with the wild type. A similar delay in callose wall breakdown was observed in the *ams* mutant, further highlighting the roles of MYB80, TEK, and AMS in this process. A mutant of *A6*, an anther-specific β-1,3-glucanase, exhibited a minor defect in breaking down the callose wall (Hird et al. 1993). Both *ams* and *myb80 tek* mutants exhibited downregulation of the five *A6* homologous genes, suggesting that MYB80 and TEK are involved in regulating *A6* genes. Additionally, the quintuple mutants of *A6* displayed a slight delay in callose degradation.

To further explore the role of A6, the authors generated two transgenic lines: pTEK::A6 in *myb80 tek* and pTEK::A6.2 in *myb80 tek*, using the *TEK* promoter to drive the expression of *A6* and *A6.2* in the *myb80 tek* background. Ectopic expression of *A6* and *A6.2* resulted in a partial restoration of callose degradation in the *myb80 tek* mutants, confirming that MYB80 and TEK regulate A6 in the tapetum as part of the tetrad callose degradation process.

Pollen exine is the outer layer that protects pollen grains from environmental stresses such as UV irradiation. Exines in the *A6* quintuple mutant (*a6-quint*) were deformed and had an unusual appearance. Pollen from *a6-quint* showed a significant reduction in both pollen germination and successful pollination when exposed to UV treatments, suggesting that the exine pattern of *a6-quint* pollen severely affects its germination and pollination process.

The authors previously demonstrated that delayed callose degradation prevents pollen abortion caused by defective exine in various photo/thermo-sensitive genic male sterility mutants (Zhu et al. 2020; Wang et al. 2022). The authors crossed the *a6 quadruple* mutant (*a6-quad*) with the *rvms-2* male sterile line, which exhibits male sterility under normal growth conditions but restores fertility at low temperatures. At lower temperatures, A6 secretion from the tapetum to the locule is also deferred, leading to delayed callose degradation and resumption of pollen development in *rvms-2* (Wang et al. 2022). The *a6-quad rvms-2* mutants showed increased fertility under normal conditions, with a significant increase in mature pollen and delayed tetrad callose degradation. These findings further strengthen that A6 plays important roles in callose degradation and pollen development.

A dual luciferase transient expression assay in *Arabidopsis* mesophyll protoplasts demonstrated that *MYB80* and *TEK* expression, driven by the *CaMV35S* promoter, induced the promoter activity of *A6* genes. The author demonstrated the binding of MYB80 and TEK to the putative sites in the A6 promoter using electrophoretic mobility shift assays and chromatin immunoprecipitation. Overall, these findings suggested that MYB80 and TEK directly regulate the expression of various *A6* homologs in the tapetum.

To further understand the temporal control of *A6* homologs, the author employed the *A9* promoter, which is specifically expressed in the tapetum during stages 5 to 9, earlier than *MYB80* and *TEK*. *A9* promoter was used to drive the expression of two *A6* homologs, *A6* and *A6.2*. The *pA9::A6.2* (wild-type) lines exhibited a more pronounced reduction in callose and showed an absence of sexine, whereas the *pA9::A6* (wild-type) lines displayed less drastic alterations. These findings indicate that A6 and A6.2 possess a unique ability of callose degradation and influence the patterning of sexine.

Overall, the study by Xu et al. (2025) investigates the role of MYB80, TEK, and A6 in callose degradation and pollen development in *Arabidopsis*. An illustration of the mechanism of action of MYB80 and TEK is provided in Fig. 1. However, the effect of the UV treatment on pollen germination and pollination could be an interesting area that can be explored further to identify the candidate genes that are less sensitive to UV irradiation during plant reproduction.
